# Medical Interpreter Training in South Korea: A Competency Alignment Analysis of Selected Certification and Curriculum Documents and a Proposed Interpreter–Nurse Pathway

**DOI:** 10.1111/inr.70218

**Published:** 2026-08-31

**Authors:** Na‐Young Kim

**Affiliations:** ^1^ Department of Russian Language and Literature Kyungpook National University Daegu South Korea

**Keywords:** competency alignment, community interpreter, Korean–Russian medical interpreter, L1, medical interpreter training, South Korea

## Abstract

**Aim:**

To conduct a document‐based competency alignment analysis of seven selected, publicly available South Korean medical interpreter certification and training documents against established international competency standards, and to propose a conceptual Interpreter–Nurse Pathway informed by this analysis.

**Background:**

Language barriers mediated by untrained personnel pose substantial threats to patient safety and health equity among linguistic minorities. The documentary articulation of internationally recognized competencies within selected South Korean certification and training materials therefore warrants systematic examination.

**Methods:**

A qualitative document analysis was performed on seven selected, publicly accessible documents comprising certification protocols, evaluative criteria, and educational syllabi. Using a benchmark of eight international competency domains, the documents were organized through an author‐developed 0–2 descriptive mapping framework that distinguishes three levels of documented integration: absence, mention without explicit instructional or assessment linkage, and explicit linkage to both instruction and assessment.

**Results:**

Within the seven documents analyzed, findings indicate a marked gap between stated competency objectives and their documented linkage to instruction and assessment. Although foundational elements such as ethics demonstrated strength, advanced technical skills—specifically remote interpreting and strategic note‐taking—lacked substantive inclusion. Only three of the eight benchmark domains (37.5%) were explicitly linked to both instruction and assessment within the selected documents (Level 2); the other five were either mentioned without such linkage (Level 1) or absent (Level 0).

**Discussion:**

The gaps identified in how the selected documents link instruction and assessment provide a basis for considering future curriculum and policy development.

**Conclusion:**

Within the documents analyzed, only three of the eight benchmark competency domains appeared in both curricular instruction and formal assessment. Of the remaining five, three were mentioned without being included in both, and two were absent. To address these gaps, this study outlines a conceptual Interpreter–Nurse Pathway as a policy and workforce‐development proposal informed by the documentary analysis.

**Implications for Nursing and health care policy:**

Specialized interpreting training in nursing curricula may offer an additional form of professional specialization for nurses with relevant linguistic proficiency.

This study proposes that policymakers consider formal interpreter–nurse pathways supported by university–hospital collaboration. The feasibility, implementation requirements, and outcomes of this proposal were not evaluated; if pursued, it would require defined professional boundaries, advanced training modules, and appropriate incentive structures for nurses with specialized linguistic proficiency.

## 1 Background

Medical interpreting has historically been categorized as a subset of community or public service interpreting (PSI) (Pöchhacker 2006, 35; Hrehovčik 2009; Krystallidou et al. 2018; Hertog and Van der Veer 2021). However, it functions as a highly specialized domain where linguistic mediation is closely linked to clinical outcomes (N. Y. Kim 2018). In contexts such as the United States, medical interpreting is framed as a fundamental service for Limited English Proficient populations (Gany et al. 2007; Gonzalez‐Barrera et al. 2024, Migration Policy Institute [MPI], Muir et al. 2025), establishing language access as a critical determinant of patient safety and health equity. Despite this, a persistent misconception remains that interpreting is a minor hurdle easily cleared by bilingual staff (Logan 2010; Al‐Yateem et al. 2023; Keller and Carrascoza‐Bolanos 2023) or ad hoc interpreters. Such practices significantly escalate the risk of misdiagnosis and adverse safety events.

The linguistic demands of the clinical encounter far exceed the parameters of general community discourse. In dermatology, for instance, terms such as “malignant melanoma,” “biopsy,” and “lesion” are commonplace (Yoo et al. 2014, 57), while cardiovascular consultations necessitate fluency in complex concepts like “coronary artery disease” and “arrhythmia.” The hazards of non‐professional mediation are illustrated by Maley (2018), who documented a case where a Bhutanese patient's “coffee‐ground emesis” was erroneously interpreted by a relative as “black cough.”

Furthermore, the conceptualization of medical interpreting as a form of community interpreting likely stems from the distinctive nature of the interpreter's role within clinical settings. It is important to distinguish the professional mandates of medical interpreters from those of conference interpreters (Código Lingua). Scholarship suggests that medical interpreters often function as intercultural mediators who navigate and reconcile cultural discrepancies, a role that offers substantial communicative advantages in healthcare delivery (Angelelli 2004b; Felberg and Skaaden 2012).

In South Korea, the 2009 amendment of the Medical Service Act drove a surge in international medical tourism, reaching approximately 500,000 patients by 2019 (Korea Health Industry Development Institute 2023). Notably, there is a substantial and growing demand from Russophone populations for health screenings and complex surgical interventions. Nevertheless, communication barriers remain the primary grievance cited by international patients (Korea Tourism Organization 2016). This dissatisfaction stems largely from the continued use of untrained staff and a lack of pre‐session briefing protocols (Hale 2007). In response, this study undertakes a document‐based competency alignment analysis of selected South Korean medical interpreter certification and training documents against international benchmarks. By establishing a baseline of eight core themes—including ethics, clinical knowledge, and remote‐interpreting protocols—the analysis examines the degree to which these competencies are operationalized within the selected documents. Furthermore, informed by this analysis, I propose a conceptual Interpreter–Nurse Pathway as a policy and workforce‐development proposal for consideration within the Korean healthcare system.

## 2 Methods

The debate over whether medical interpreting should be conceptualized as a standard extension of community‐based linguistic mediation or as a standalone profession requiring rigorous clinical proficiency remains a central tension in the field. This controversy is fundamentally rooted in the high degree of specialization intrinsic to the healthcare environment. As established in the preceding sections, the discourse within clinical encounters and institutional collaborations demands not only precise terminological mapping but also advanced cognitive skills, such as strategic note‐taking and interactional management.

Given this backdrop, the present study adopts a document‐based approach to seven selected South Korean medical interpreter certification and training documents. The analysis examines how these documents articulate institutional expectations for training and assessment and assesses their alignment with the international competency domains used as the benchmark in this study. Accordingly, the findings concern the selected documents rather than practitioner competence or system‐wide practice. The study therefore addresses the following research question:

RQ: To what degree do the seven selected South Korean medical interpreter certification and training documents align with—or diverge from—the international competency domains used as the benchmark in this study?

### 2.1 Global Benchmarks and the Analytical Framework

The purpose of this section is to define the theoretical benchmarks and core themes used to evaluate how the seven selected South Korean medical interpreter certification and training documents align with—or diverge from—the international competency domains adopted in this study.

In selecting a primary benchmark, this study considered various international frameworks, including Australia's NAATI (National Accreditation Authority for Translators and Interpreters) and the ISO 13611 standard (International Organization for Standardization 2024). NAATI offers a pioneering model for national accreditation and social integration of interpreters, while ISO 13611 provides general quality guidelines for service delivery. However, the US policy framework was ultimately prioritized due to its distinct emphasis on medical interpreting as a safety‐critical clinical profession rather than a general social service. The US model is supported by extensive empirical evidence linking qualified interpretation to the mitigation of diagnostic errors and improved patient outcomes. This clinical‐centric approach is particularly relevant to the South Korean context, where the medical tourism profile—marked by an influx of Russian‐speaking patients seeking complex surgical interventions and advanced screenings—necessitates high‐precision communication in high‐stakes clinical encounters. Consequently, the US standards (National Council on Interpreting in Health Care 2005) serve as a reference point for examining how the selected South Korean certification and curriculum documents represent the competencies required for high‐stakes clinical interpreting.

Drawing from these international standards and prior literature (Angelelli 2004a; Hsieh 2010; Bot and Verrept 2013), eight core themes were identified as the benchmark framework: (1) Ethics, confidentiality, and neutrality; (2) Accuracy, completeness, and terminological competence; (3) Patient–provider communication (e.g., plain language, teach‐back); (4) Clinical knowledge and medical terminology; (5) Note‐taking, memory, and strategic skills; (6) Remote interpreting (VRI/OPI) protocols; (7) Vulnerable populations and patient safety; and (8) Assessment competence (rubrics and feedback design).

Ethical and terminological proficiency remain fundamental to patient safety. The following sections apply an author‐developed 0–2 descriptive mapping framework to examine how each of the eight benchmark themes is represented in the seven selected South Korean certification and training documents.

### 2.2 Current Status and Characteristics of South Korea's Medical Interpretation Market

This section delineates the selection criteria for the documents analyzed and the scoring protocols used to evaluate their alignment with the eight core themes. The infrastructure for medical interpreting in South Korea underwent a transformative shift following the 2009 amendment of the Medical Service Act. By 2019, the number of inbound patients reached approximately 500,000, driven in large part by the high accessibility and clinical quality of Korea's health screening services. Notably, Russian‐speaking patients accounted for nearly 30,000 visits in 2019 alone, with a substantial proportion seeking complex diagnostic and surgical interventions.

These market dynamics necessitate a level of interpreter competency that goes beyond basic linguistic mediation. The prevalence of outpatient‐centered care, combined with the continued reliance on untrained internal staff, underscores the urgency of assessing the extent to which the eight core themes are operationalized as concrete evaluative tools within the seven selected documents. For this analysis, seven publicly available documents concerning South Korean medical interpreter qualification and training were selected, as detailed in Table 1.

**TABLE 1:** Selected South Korean medical interpreter certification and training documents.

Category	Document title	Source institution	Brief description
Certification (*n* = 3)	Medical Interpreter Qualification Examination Guidelines	KOHI	Outlines exam domains, scoring criteria, and application protocols.
	Scoring Rubrics for Oral Performance Examination	KOHI	Specifies evaluative metrics for accuracy and terminological proficiency.
	Official Candidate Handbook and Assessment Criteria	KOHI	Provides procedural instructions and baseline assessment standards.
Education (*n* = 4)	Professional Medical Interpreter Training Program Syllabus	KOHI	200‐hour comprehensive module‐based instructional design.
	Advanced/Supplementary Medical Interpreter Training Course	KOHI	Specialized curriculum for continued professional development.
	Representative Undergraduate Syllabus A: Medical Interpreting	University A	Core pedagogical guide for language majors entering the field.
	Representative Undergraduate Syllabus B: Medical Interpreting	University B	Comparative syllabus focusing on clinical terminology and skills.

*Sources*: Korea Human Resource Development Institute for Health and Welfare (n.d.a, n.d.b, 2017a, 2017b, 2024a, 2024b), Sungshin Women's University (n.d.), and Kyungdong University (n.d.).

Each document was evaluated using an author‐developed, three‐tier Level of Operationalization scale—a descriptive mapping framework devised specifically for this study:

- Score 0 (Absent): No reference to the competency or related modules exists within the documentation.
- Score 1 (Mentioned): The competency is acknowledged as a requirement, yet the document lacks a specific methodology for instruction or assessment.
- Score 2 (Operationalized): The competency is explicitly linked to a curricular module and a corresponding assessment criterion, indicating documented integration across instruction and assessment.

To apply this descriptive framework consistently, I established explicit decision rules for borderline cases, particularly when a competency was articulated in pedagogical objectives but lacked corresponding assessment criteria. A theme was mapped to Level 2 (Operationalized) only when the documents explicitly linked instructional delivery with formal assessment. When a competency was nominally included in a syllabus but was not linked to both a discrete instructional module and an explicit assessment criterion, it was mapped to Level 1 (Mentioned). These categories were used to organize the documentary evidence descriptively and were not treated as a validated measurement scale. For instance, “Patient–Provider Communication” (Theme 3) and “Vulnerable Populations” (Theme 7) were cited as learning objectives in several curricula; however, their lack of integration into official certification rubrics as measurable performance items resulted in their classification at Level 1.

To examine the consistency with which I applied the descriptive mapping rules, I repeated the coding after a two‐week interval and compared the second set of assignments with both the initial coding and the source documents. No discrepancies were identified between the two rounds. This agreement indicates that I applied the mapping rules consistently on the two occasions; however, because both rounds were conducted by the same researcher, it does not establish interrater reliability, eliminate researcher subjectivity, or demonstrate reproducibility by independent coders. The procedure was therefore treated solely as an internal consistency check. For example, the “Ethics” theme was mapped to Level 2 (Operationalized) because it is explicitly defined as a standalone written examination domain with corresponding professional conduct rubrics. Conversely, ‘Note‐Taking Strategies’ was mapped to Level 0 (Absent) because the selected documents contained no documented instructional modules or formal assessment criteria for this theme. The specific mapping results and corresponding qualitative rationales are detailed in the subsequent Results section.

### 2.3 Korean and American Medical Interpreter Training Process

**FIGURE 1:**
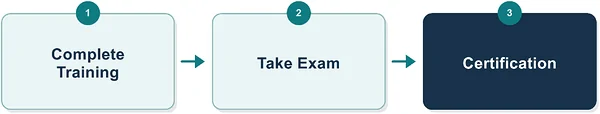
American medical interpreter training process.

**FIGURE 2:**
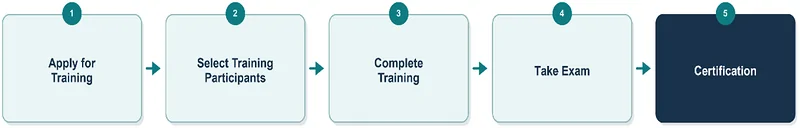
Training and certification pathway described in the selected South Korean documents.

Figures 1 and 2 provide an overview of the medical‐interpreting education and certification pipelines in the United States (international benchmark) and South Korea (evaluation target), respectively. The purpose of this section is not to conduct a simplistic cross‐national comparison of outcomes but to establish the structural context necessary for interpreting the subsequent alignment analysis (RQ). Specifically, it provides institutional background for the seven documents analyzed, thereby supporting interpretation of how the selected Korean certification and training documents align with—or diverge from—the benchmark domains used in this study.

In the United States, medical‐interpreting education is predominantly facilitated by private organizations, where the financial responsibility for training lies primarily with the individual participant. These programs typically utilize open‐enrollment policies requiring only basic prerequisites, such as age or general education markers, which grants institutions considerable programmatic autonomy. However, this decentralized, market‐driven structure results in significant heterogeneity across the sector, particularly in how contact hours, instructional modules, and evaluative protocols are documented and standardized.

In contrast, medical‐interpreting education in South Korea is organized around a centralized, selection‐oriented pipeline supported by public funding and state‐led oversight. The documents analyzed here articulate this structure through formal screening prior to admission and through publicly accessible evaluation domains and scoring criteria. What such documents can be examined for is not the practitioner competence this pipeline secures in practice, but the coherence with which they link curricular elements to learning outcomes and performance‐based assessment criteria.

This funding model, while reinforcing equity and accessibility, can also constrain institutional flexibility for innovation and experimentation. Consequently, how Korean documents address the assessment competence theme—specifically the use of rubrics, practical evaluations, and post‐assessment feedback—warrants close attention in determining alignment with international standards.

This study does not overinterpret these structural differences as causal factors in alignment outcomes. Instead, it evaluates the extent to which the seven selected Korean documents represent and link the eight core themes to instruction and assessment. The next section presents the aggregate 0–2 mapping results for the eight benchmark themes across the seven selected documents and explains the documentary rationale for each assigned score.

## 3 Results

### 3.1 Current Status of Russian Medical Interpreter Education in South Korea

One of South Korea's leading medical interpreter education institutions is the Korea Human Resource Development Institute for Health and Welfare, established under the Ministry of Health and Welfare of the Republic of Korea (You 2020). KOHI has developed and actively implemented programs to cultivate qualified experts for the medical‐interpreting field. The professional training course consists of 200 hours of instruction—held eight hours every Saturday over six months—to prepare practitioners for the clinical field. The program represents a significant public investment, with the total cost of approximately USD 7,200 per participant being fully subsidized. This includes USD 6,000 in direct government support alongside an additional USD 1,200 covered by the Korea Human Resource Development Institute for Health and Welfare (KOHI 2024a). Afterward, students prepare for the medical interpretation examination through programs such as an additional course for medical interpretation (KOHI 2017a, 2017b).

However, despite substantial public support for tuition, formal Korean–Russian medical interpreter training was limited in scale in 2018, when KOHI selected approximately ten trainees per language, including Russian (S. M. Kim 2018).

Available evidence suggests that completion of formal KOHI training was not widespread among the surveyed Russian‐language medical interpreters. Of the 218 medical interpreters examined in Kim's (2015) study, 9.2% had completed the KOHI medical interpreter training program.

Some interpreters appear to have developed their Russian–Korean medical‐interpreting competence through self‐directed workplace practice. Kim (2017), for example, notes that some Koryo‐Saram interpreters working mainly in medical settings had developed their competence through several years of practice.

Additionally, Kim (2017) describes the Russian‐language medical interpreters working in the Korean clinical settings examined in that study as predominantly Koryo‐Saram. Koryo‐Saram refers to those who migrated from the Korean peninsula to the coastal region because of the difficult living conditions at the end of the Joseon Dynasty. They overcame the difficulties in their new settlements and built their own diaspora to lead a stable life. Later, in 1937, the Soviet leadership's shifting policies led to the forced migration of Koryo‐Saram to Central Asia (Lee 2003). Those currently working as medical interpreters in Korea typically belong to the fourth or fifth generation of this diaspora. Russian is reported to be the L1 of many of these interpreters (Kim 2017). Under KOHI's published eligibility criteria, applicants were eligible only if their L1 was either Korean or the language of the track to which they applied (KOHI 2017b; KOHI 2024c). For the Russian‐language track, applicants whose L1 was Uzbek or Kazakh were therefore not explicitly included in the program's standard eligibility categories, even if they were proficient in Russian.

In Korean clinical settings, medical staff generally communicate diagnostic and clinical information in Korean. For Russian‐L1 interpreters, this discourse is processed as B‐language input. When Korean B‐language proficiency or specialized medical‐interpreting training is insufficient, incomplete comprehension of the source message may lead to inaccuracies in the Russian target‐language rendition. Kim (2017) suggests that Russian‐L1 interpreters’ relative ease in communicating in Russian may help reduce psychological distance and facilitate rapport with patients during pre‐consultation interactions.

Another problem is that the medical‐interpreting job market is an open market, and people with or without prior medical interpretation education can apply for positions in this field. KOHI provides medical interpreter training and the medical interpreter qualification tests, but these trainings and exams are only recommendations and are not considered compulsory training or qualification exams. In addition, although the medical interpreter qualification tests include clinically relevant knowledge domains, their evaluation criteria also cover areas that are not central to interpreters’ routine responsibilities in clinical settings; the overall balance of the assessments therefore warrants refinement. Table 2 shows the evaluation criteria of the medical interpreter competency test.

**TABLE 2:** Evaluation criteria of the medical interpreter qualification test in South Korea.

Examination domain	Subdomain	Key content and examples
1. Medical Service	1‐1 Local healthcare culture	Health care system, etc.
	1‐2 Medical interpretation ethics	Medical ethics, etc.
2. Clinical Medical Service	2‐1 Understanding medical procedures	Medical treatment, examination, etc.
	2‐2 Patient education	Hospitalization/discharge education, drug consultation, emergency management, etc.
3. Hospital system	3‐1 Hospital administration	Hospital affairs, customer management, medical information management system, etc.
	3‐2 Medical law	Medical law, medical dispute mediation law, etc.
4. Basic Medicine	4‐1 Medical terms	Medical term component
	4‐2 Anatomy and Physiology	General terms of human tissues and systems (human body and disease)
	4‐3 Clinical Terminology	Medical terms categorized by specific medical procedures

*Note*: This table summarizes the official evaluation framework administered by the Korea Human Resource Development Institute for Health and Welfare (KOHI).

*Source*: Korea Human Resource Development Institute for Health and Welfare (2024a).

Table 2 confirms that the medical interpreter competency test includes medical terms, anatomy and physiology, and clinical terminology. The concern therefore lies not in the absence of a medical‐terminology component but in the overall balance and construct validity of the evaluation criteria. To improve field readiness, the assessment could place greater emphasis on the interpreting competencies most frequently used in clinical practice.

### 3.2 Competency Alignment Results (Based on the Eight‐Theme Benchmark)

Using a benchmark of eight international competency domains, the documents were organized through a 0–2 descriptive mapping framework developed specifically for this study. The framework distinguishes three levels of documented integration: absence, mention without explicit instructional or assessment linkage, and explicit linkage to both instruction and assessment:

- Level 0 (Absent): No discernible evidence of the theme within the analyzed documents, such as examination announcements or curricular outlines.
- Level 1 (Mentioned): The theme is nominally included in learning objectives or the examination scope, yet lacks dedicated instructional hours (modules) or explicit evaluative criteria (rubrics), indicating a state of “superficial alignment.”
- Level 2 (Operationalized): The theme is explicitly represented in a curricular module and linked to a corresponding assessment criterion; within this author‐developed framework, this documentary pattern is categorized as “deep alignment.”

To make the evidential basis for the mapping decisions transparent, the analysis relied on explicit textual indicators in the source documents, such as standalone modules, specific credit allocations, and designated instructional hours. As described in Section 2.2, the repeat‐coding procedure was treated solely as an internal consistency check. It does not establish interrater reliability, eliminate subjective judgment, or demonstrate reproducibility by independent coders. The author‐developed framework was used descriptively to distinguish “what is taught” from “how competencies are institutionalized and assessed.”

Table 3 provides a consolidated overview of the operationalization scores assigned to each of the eight core themes across the seven selected documents. Detailed rationales for each assigned score are provided in the subsequent qualitative analysis.

**TABLE 3:** Summary of operationalization scores and alignment levels for the eight core international benchmarks.

No.	International competency theme	Score (0–2)	Alignment status
1	Ethics, Confidentiality, and Neutrality	2	Operationalized
2	Accuracy, Completeness, and Terminological Competence	2	Operationalized
3	Patient–Provider Communication	1	Mentioned
4	Clinical Knowledge and Medical Terminology	2	Operationalized
5	Note‐Taking, Memory, and Strategic Skills	0	Absent
6	Remote Interpreting (VRI/OPI) Protocols	0	Absent
7	Vulnerable Populations and Patient Safety	1	Mentioned
8	Assessment Competence	1	Mentioned

The systematic application of this descriptive framework to the seven selected documents produced the following findings, categorizing each core theme according to its documented level of alignment:

Ethics, Confidentiality, Neutrality (Score: 2). Written exam domains include medical‐interpreting ethics, and curricula emphasize professional ethics and confidentiality. This demonstrates that ethical principles, confidentiality, and neutrality are treated as essential competencies across both education and assessment, reflecting a high degree of alignment.

Accuracy, Completeness, Terminological Competence (Score: 2). The selected documents identify the accurate rendering of information and domain‐specific terminology as primary evaluation targets. Oral examinations prioritize accuracy and coherence; written materials and curricula cover health services, clinical knowledge, and medical terminology extensively. This finding aligns with international standards requiring accurate and complete rendition without additions, omissions, or substitutions, and with evidence that interpreting errors and omissions may have clinical consequences (National Council on Interpreting in Health Care 2005; Flores 2005).

Patient–Provider Communication (plain language, teach‐back, interaction management) (Score: 1). While the curricula acknowledge the importance of communication between clinicians and patients, there is limited evidence that plain language strategies, teach‐back, and interaction management are structured as standalone modules or specific assessment items. Compared with the interaction‐based models described in the international literature (Hsieh 2006; Hsieh and Kramer 2012; Felberg and Skaaden 2012), the selected documents provide only partial evidence of alignment for this theme (Score 1).

Clinical Knowledge and Medical Terminology (Score: 2). From basic biomedical concepts to anatomy, diseases, tests, and procedures, a broad range of clinical knowledge and terminology appears in both written exams and curricula. Oral examinations also probe interpreters’ understanding of clinical contexts. This represents a strongly aligned area.

Note‐taking, Memory, and Strategic Skills (Score: 0). No explicit modules or assessment criteria for note‐taking or memory strategies (e.g., chunking and summarizing) were found in the selected documents. Accordingly, this theme was mapped to Level 0 (Absent).

Remote Interpreting (VRI/OPI) Operations and Protocols (Score: 0). Documents do not specify procedures or security guidelines tailored to video (VRI) or phone (OPI) medical interpreting—e.g., equipment setup, pre‐session protocols—or corresponding assessment bases. Given the post‐COVID surge in demand and importance worldwide, this theme constitutes a notable gap in the selected documents.

Vulnerable Populations, Patient Safety, and Equity (Score: 1). Items such as the rights and responsibilities of foreign patients and the prevention of medical incidents/disputes are mentioned, but specialized responses for linguistically vulnerable groups (children, older adults, persons with disabilities, etc.) and case‐based training/assessment remain limited. Implementation is therefore partial (score = 1).

Assessment Competence (rubrics, scenario‐based performance, feedback) (Score: 1). The certification adopts a two‐stage structure (written + oral) with scenario‐based oral components and publishes pass/fail thresholds, ensuring a degree of transparency. However, detailed scoring rubrics and feedback procedures remain undisclosed, meaning the sophistication of assessment design is only partially aligned (score = 1).

To summarize, the eight‐theme scores for the selected document set totaled 9 of a possible 16 points (56.3%). Six of the eight themes (75%) were represented at least at Level 1, and three themes (37.5%) met the Level 2 criterion of explicit linkage to both instruction and assessment. Ethics, confidentiality, and neutrality; accuracy, completeness, and terminological competence; and clinical knowledge and medical terminology were mapped to Level 2. The remaining five themes—patient–provider communication; note‐taking, memory, and strategic skills; remote interpreting; vulnerable populations, patient safety, and equity; and assessment competence—were mapped to Levels 0 or 1. Building on these documentary findings, Section 4 presents proposals for future curriculum and workforce development in South Korea.

### 3.3 Implications of the Alignment Results

The descriptive mapping results in Section 3.2 may be interpreted in relation to the workforce context discussed in Section 3.1. In particular, explicit training in patient–provider communication and note‐taking and memory strategies may be relevant for interpreters who process complex Korean clinical discourse as an additional‐language input.

From this perspective, Theme 3 (Score 1) and Theme 5 (Score 0) indicate that the selected documents do not fully operationalize these competencies through explicit instruction and assessment. This constitutes a documentary gap in competencies relevant to clinical accuracy; the analysis does not establish that the gap produces patient‐safety outcomes in practice or that it represents a system‐wide failure.

In summary, the selected documents do not fully specify the pedagogical mechanisms through which these competencies would be taught and assessed. The documentary findings therefore support considering the following priorities: (i) refining assessment practices through public rubrics, scenario‐based practical assessments, and feedback; (ii) introducing remote‐interpreting protocols; and (iii) developing explicit modules for patient–provider communication and note‐taking and memory strategies. Additional proposals concerning vulnerable populations, patient safety, and workforce‐pipeline development are presented in the next section as directions for future curriculum and policy development rather than as empirically validated interventions.

## 4 Discussion

The preceding alignment analysis identified strengths in ethics, accuracy, and clinical knowledge across the seven documents analyzed, together with limited or partial operationalization in assessment design, remote interpreting, fine‐grained patient–provider communication strategies, note‐taking and memory strategies, and vulnerable populations and safety. Because these findings concern the selected documents and cannot be taken to represent South Korea's medical interpreter infrastructure in its entirety, the proposals advanced in the remainder of this section are conceptual ones, developed along two axes: (i) the redesign of instructional modules and (ii) the expansion of workforce‐development pathways. The feasibility and effects of these proposals were not empirically evaluated in this study.

### 4.1 Education for Russian Majors Whose L1 Is Korean

In South Korea, there are about 25 universities nationwide offering Russian or Russian‐related majors (Career Net 2023). The employment rate of Russian majors is about 60%, which is better than other majors in the humanities field. Specifically, Career Net data show that Russian Language and Literature departments lead with an employment rate of 61.8%, followed by English (61.4%), Korean (57.5%), History (56.9%), and Philosophy (56.1%).

Starting salaries for Russian majors are also notably higher, averaging $1904—significantly exceeding those of graduates in English ($1399), Philosophy ($1377), History ($1341), and Korean ($1312). This is because being proficient in Russian offers a distinct advantage. In South Korea, Russian is considered a unique foreign language and also carries significant geopolitical weight, which likely boosts the employability and salary prospects of these graduates.

However, medical interpreting could provide an additional specialized career pathway for Russian‐language graduates seeking to broaden their employment opportunities. Also, as mentioned earlier, having L1 Korean language skills can be advantageous for medical interpreters. Although Russian‐speaking interpreters could provide a sense of psychological stability, the most important element of medical interpreting is accurate interpretation. Therefore, if medical‐interpreting education were integrated into Russian‐language programs at the undergraduate level, these graduates—who might otherwise have difficulty entering the workforce—would gain access to a wider range of career opportunities. There are organizations where medical interpretation courses have been newly established and taught at the undergraduate level, but these remain few in number. Only about 12% of all Russian‐related departments have medical interpretation courses (Career Net 2023).

Given this limited provision, medical‐interpreting education could be considered by universities seeking to diversify the career pathways available to students in their Russian‐language programs, particularly institutions that can support university–hospital collaboration and supervised practicum opportunities. Furthermore, given the geographic proximity between the Korean Peninsula and Russia, any improvement in bilateral relations may increase demand for medical services among patients from Vladivostok and other maritime regions.

### 4.2 Medical Interpreter Training for Prospective Nurses

Nurses are often the primary point of contact for patients within the clinical environment. Nurses have reported that interpreters are necessary when communicating with patients who do not speak the dominant language (Rifai et al. 2018), and bilingual nurses may consequently be asked to perform interpreting functions (Fernandez 2014). However, bilingual clinical status does not in itself establish interpreting competence. In Elderkin‐Thompson et al.’s (2001) study of 21 physician–patient encounters mediated by nurses who had not been trained as medical interpreters, approximately half contained serious miscommunication that affected either the physician's understanding of the patient's symptoms or the perceived credibility of the patient's concerns. The findings indicate that clinical familiarity may facilitate communication but can also lead nurses to render information in accordance with clinical expectations rather than the patient's actual account. Dual‐role practice therefore requires specialized interpreter training and explicit institutional safeguards rather than reliance on bilingual ability or clinical status alone.

Russian‐language specialization may constitute one possible professional pathway for nurses working in international medical settings. This possibility is relevant because numerous Korean hospitals listed in the national Medical Korea directory maintain dedicated international healthcare centers and multilingual services for foreign patients (Korea Health Industry Development Institute 2023). These centers serve foreign patients who are not covered by the Korean national health insurance system and apply an international fee structure within an administrative framework distinct from that generally available to Korean citizens (Ministry of Health and Welfare 2019).

Previous studies have examined the interactional roles performed by medical interpreters and other bilingual mediators across clinical contexts (Kim 2017; Cerezo‐Herrero and Shaffer 2021; Slusarz 2024; Bamford et al. 2024; Ono and Yang 2024; Vallati 2025). However, this literature should not be taken to establish that nurse‐provided interpreting is generally advantageous. Research focused specifically on dual‐role nurses indicates that potential communicative benefits can coexist with patient‐assignment disruption, increased workload, time taken away from nursing duties, missed or misinterpreted information, and perceived clinical risk (Villanueva 2023; Chang et al. 2021). Professional medical interpreting requires technical expertise and the capacity to coordinate complex clinical interactions (Hsieh 2010; Wu and Rawal 2017). Clinical knowledge may support accurate comprehension of medical terminology and procedures, but it neither establishes interpreting competence nor authorizes the interpreter–nurse to independently explain, omit, or modify either party's message. This distinction strengthens the case for professional medical‐interpreting education in nursing colleges. Russian is offered as a general‐education or second‐foreign‐language subject at some Korean universities. For example, Yonsei University's (2025) undergraduate course guide includes the College of Nursing within its first‐year general‐education framework and lists Russian I–III and Russian Practice I among its second‐foreign‐language offerings. However, such courses are introductory rather than clinically specialized: Korea University's (n.d.) Russian I course description, for example, focuses on pronunciation, basic grammar, simple vocabulary, and sentence‐pattern practice. Accordingly, these general‐education offerings alone are insufficient preparation for medical interpreting.

Therefore, it might be advantageous to teach specialized foreign‐language education at nursing colleges and foster nurses specializing in Russian. It is also understood that the nursing curriculum is extensive, and the employment rate of nurses in the Korean job market is quite high (about 85%, Higher Education in Korea n.d.). Hence, the motivation for professional foreign‐language education may not be strong among Korean nursing students. However, because of excessive workload and organizational culture, the turnover rate of nurses is also very high. The turnover rate for new nurses is quite high and has continued to rise, reaching 57.4% in 2022 (Ministry of Health and Welfare 2018, Hospital Nurses Association n.d.). Against this workforce background, the proposed pathway cannot be regarded as an established response to turnover or burnout. Nevertheless, as an optional form of professional specialization, it may broaden the professional‐development opportunities available to prospective nurses with the requisite linguistic proficiency.

However, the specialized foreign‐language and interpreting competence required in medical institutions is unlikely to be developed through short‐term instruction. A standardized comparison found that trained medical interpreters were substantially less likely than untrained interpreters to make clinically significant errors (Gany et al. 2010), and an approximately 80‐hour medical interpreter education program was associated with significant gains in written and oral assessment performance (Hasbún Avalos et al. 2013). Flores et al. (2012) further found that, among professional interpreters, those with at least 100 hours of prior training committed fewer errors than those with less training and suggested that 100 hours might serve as a useful minimum. Taken together, these findings provide a rationale for considering sustained rather than short‐term instruction in nursing colleges. The prevailing model of limited liberal arts language study is insufficient for clinical specialization; instead, high‐demand languages like Russian could be integrated into the nursing curriculum through a structured, multi‐year minor or optional major track.

### 4.3 Pros and Cons of Medical Interpreter Training for Learner Types

Based on the preceding discussion, the comparative advantages and challenges of Russian‐language medical interpreter education for Russian majors and prospective nurses can be summarized in Table 4 as follows:

**TABLE 4:** Potential advantages, challenges, and required safeguards of the proposed training pathways.

Learner type	Potential advantages	Challenges and required safeguards
Russian Majors	Prior study of Russian language and culture Korean L1 proficiency that may support comprehension of Korean clinical discourse An additional professional specialization and career pathway	Need for advanced Russian proficiency Need for clinical knowledge and specialized interpreting skills Need for supervised practicum and competency‐based assessment No guarantee of interpreting accuracy based on academic major or L1 profile alone
Prospective nurses	Existing clinical knowledge that may support comprehension of medical terminology and procedures Familiarity with clinical settings and workflows An optional professional specialization for nurses with the requisite linguistic proficiency	Need for sustained advanced‐language and interpreting training Workload and divided attention Role conflict and professional‐boundary risks Confidentiality, neutrality, and informed‐consent concerns Need for protected interpreting time and independent interpreter backup

### 4.4 Recommendations Informed by the Alignment Results

Drawing on the alignment analysis and the preceding situational discussion, this section sets out conceptual curriculum‐redesign and workforce‐development proposals for Russian majors and pre‐service nurses.

#### 4.4.1 Curriculum Redesign: Russian Majors

Establishing or expanding a medical‐interpreting track within undergraduate Russian programs could be considered. Given the current scarcity of such courses across Russian‐related departments, such a track may enable students to acquire systematic training in domain terminology, foundational clinical knowledge, and situation‐specific interpreting techniques during their studies. This strategy would facilitate the integration of professional competencies directly into the undergraduate curriculum. Leveraging the strengths of Russian majors whose L1 is Korean—namely, rapid grasp of Korean medical terminology and accurate delivery—the following module bundle could be considered:

Introduction to Medical Interpreting (roles, ethics, and boundaries); Medical Terminology & Clinical Foundations (core content by specialty); Patient–Provider Communication Strategies (plain language and teach‐back); Note‐Taking and Memory Strategies (structured note‐taking); Remote Interpreting (VRI/OPI) operations and protocols; Interpreting for Vulnerable Populations and Patient Safety (Theme 7); Scenario‐Based Practicum with Rubric‐Based Assessment and Feedback.

Each module would need to move beyond mere knowledge transfer to focus on cultivating practical competencies that reflect the complexities of real‐world clinical environments. Syllabi could explicitly define learning outcomes (LOs) and illustrate their alignment with instructional and assessment methods, such as rubrics, checklists, and feedback procedures. This may offer a mechanism for transitioning themes that scored lower—including Theme 3 (communication), Theme 5 (note/strategy), Theme 6 (remote), Theme 7 (vulnerability/safety), and Theme 8 (assessment competence)—from absence or superficial mention (scores = 0 or 1) to full operationalization (score = 2).

#### 4.4.2 Workforce‐Pipeline Expansion: Russian‐Major Pathway

A systematic approach would be required to integrate Russian majors into the medical‐interpreting talent pool. Through university–hospital linkages, a staged pathway could be built:

Pre‐selection (language/aptitude screening) → Completion of the foundational track (modules above) → On‐site practicum at international medical/check‐up centers (with mentoring) → Micro‐credentials (completion certificates/competency badges) → Certification‐exam preparation → Placement (employment/freelance matching).

Cooperation arrangements such as memorandums of understanding among universities, hospitals, and local governments are proposed as a possible structure for placements tailored to Russian/CIS demand. The model is intended to build on the documentary strengths identified in Themes 2 and 4 and to increase curricular attention to the lower‐scoring themes.

#### 4.4.3 Curriculum Redesign: Pre‐Service Nurses

Establishing or enhancing a specialized medical‐interpreting track within nursing curricula could likewise be considered. Current general‐education foreign‐language offerings (e.g., Russian) could be upgraded to the professional level to build cumulative, clinically usable language and interpreting competencies. Possible modules are:

Introduction to Medical Interpreting (role definitions, confidentiality, neutrality, professional boundaries, and the management of workload, divided attention, role conflict, and informed‐consent encounters in dual‐role practice); Clinical Knowledge and Medical Terminology (core procedures, medications, and consent terminology by specialty); Patient–Provider Communication Strategies (plain language, teach‐back, mediation); Note‐Taking and Memory Strategies; Remote Interpreting (VRI/OPI) operations, security, and pre‐session protocols; Vulnerable Populations, Patient Safety, and Equity (cases involving children, older adults, persons with disabilities, and patients with limited health literacy); Scenario‐Based Practicum (OSCE‐type) with Rubric‐Based Assessment and Feedback.

Each module would need to be designed so that LOs, instruction, and assessment are explicitly linked in documentation, with clinical practicums incorporating routine role‐play/simulation. As a provisional curriculum‐design proposal, the curriculum would allocate 100 hours across intensive practicums and microlearning (short, repeated simulations). Although the existing state‐led vocational program (KOHI 2024b) mandates 200 hours for practitioner specialization, the proposed 100‐hour allocation is an evidence‐informed but provisional benchmark for undergraduate curriculum planning: Flores et al. (2012) identified ≥100 hours of prior training as the cut point most clearly associated with fewer total errors and fewer errors with potential clinical consequences among 20 professional interpreters, while its suitability for undergraduate nurse–interpreter education requires prospective evaluation. This approach may offer a practical foundation for transitioning themes with lower alignment scores—specifically themes 3, 5, 6, 7, and 8—from absence or superficial mention (scores = 0 or 1) to full operationalization (score = 2).

#### 4.4.4 Workforce‐Pipeline Expansion: Interpreter–Nurse Pathway

As one component of this workforce‐development proposal, the following staged pathway is outlined for pre‐service and in‐service nurses who may seek specialization as medical interpreter–nurses:

1. Pre‐selection (foreign‐language proficiency and clinical‐communication aptitude),
2. Bridge‐training completion (medical‐interpreting track above),
3. On‐site practicum at international/check‐up centers (with mentoring and pre‐/debriefing),
4. Micro‐credential issuance (competency badges/certificates),
5. Certification‐exam preparation and scenario re‐assessment,
6. Hospital placement/dedicated assignment or registration in a remote‐interpreting pool.

To facilitate the practical integration of this pathway, hospitals would need to formalize administrative support systems, including language differentials (stipends/grade adjustments), dedicated interpreting slots in duty rosters, and standard pre‐session information‐sharing forms along with post‐session feedback/QA procedures. Specifically, to mitigate dual‐role conflicts between patient care and interpreting, guidelines would need to define role boundaries and escalation/mediation criteria. Furthermore, maintaining a remote‐interpreting roster would be required to address the gap in Theme 6 (Remote Interpreting Operations) and respond effectively to night and emergency demands. By integrating nurses’ clinical strengths with Themes 2 and 4, the proposed pathway is designed to address the domains that received lower mapping scores (Themes 3, 5, 6, 7, and 8).

#### 4.4.5 Curricular Integration and Implementation Strategies: Navigating Pedagogical Constraints

To transition the Interpreter–Nurse Pathway from a theoretical proposition to a viable educational reality, the project would need to address the structural rigidities of the already saturated nursing curriculum. To maximize both flexibility and feasibility, this study proposes a staged integration strategy as follows:

- Modular Certification via Micro‐Credentials: Rather than attempting to compress the expansive medical‐interpreting syllabus into a single credit‐bearing course, a modular certification system utilizing micro‐credentials is proposed. By deconstructing the curriculum into targeted units—such as Medical‐Interpreting Ethics, Russophone Clinical Terminology, and Interactional Management—institutions can issue digital badges or partial certifications for each milestone. This distributed learning model is intended to distribute training across smaller units and support personalized competency development; whether it reduces cognitive overload or improves competency acquisition would require empirical evaluation.
- Immersion through Vacation Intensives: To bypass the academic density of the regular semester, intensive 4‐ to 6‐week programs could be established during summer and winter recesses. These intensives may foster high‐immersion environments that accelerate linguistic and technical proficiency, offering a pragmatic alternative that avoids encroaching on the core nursing credits required during the term.
- Specialized Tracks and Dual‐Degree Models: For students seeking a definitive professional path in medical mediation, undergraduate programs could implement specialized Global Nursing‐Medical‐Interpreting tracks or dual‐degree models. Formally documenting this specialization on the degree could enhance graduates’ professional competitiveness in the healthcare labor market.
- Organic Integration with Clinical Practicum: A more seamless implementation involves allocating a portion of mandatory clinical hours to assignments within international clinics or dedicated foreign patient screening centers. This can help ensure that interpreting training does not remain an isolated academic exercise but is instead integrated into clinical encounters in real‐world healthcare settings.

By adopting this modularized framework, nursing colleges can cultivate a specialized workforce responsive to evolving healthcare demands without diluting the pedagogical rigor of the core nursing curriculum.

### 4.5 Implications for Nursing and Health Policy

The documentary gaps identified in the alignment analysis inform the conceptual development of a specialized interpreter–nurse workforce proposal. Because nurses are frontline clinicians, their potential linguistic roles warrant consideration in relation to patient safety and clinical communication. The pathway is also presented as a possible workforce‐development option for nurses seeking specialized professional roles; whether it affects burnout, attrition, workforce retention, patient safety, or clinical outcomes remains a question for future empirical research.

To transition this proposal from a conceptual model to an operational reality, the following three policy‐level mechanisms are proposed:

- Institutionalized Credentialing and Licensure Endorsements: Operationalizing this pathway would require a credentialing framework. Drawing on the Qualified Bilingual Staff model used by Kaiser Permanente (2013) and the statutory mandates of California's SB 853 (Health Care Language Assistance Act 2003), South Korea could consider a national Medical Interpreter–Nurse Endorsement. Under this provisional curriculum‐design proposal, the endorsement would include a suggested cumulative training allocation of 100 hours together with competency‐based assessment.
- Structural Compensation and Accreditation Incentives: The viability of the interpreter–nurse model would depend on formal compensation mechanisms that recognize the added value of bilingual clinicians. Policymakers could integrate certified interpreter–nurse staffing into hospital accreditation standards to provide a fiscal incentive for institutions. Furthermore, language‐based compensation differentials—such as tiered stipends or grade adjustments—are proposed as a means of formally recognizing the additional responsibilities associated with certified interpreter–nurse practice.
- Operational Governance and Role‐Switching Protocols: Dual‐role interpreter–nurse practice creates distinct professional and ethical risks because a single practitioner remains accountable for two roles with different immediate priorities. Research on certified dual‐role nurses reports patient‐assignment changes, frustration, and time taken away from nursing duties (Villanueva 2023). In a qualitative study of 12 bilingual nurses in two emergency departments, nurses were recruited for ad hoc interpreting under clinical urgency and workflow pressure; interpreting increased their workload and was associated with missed or misinterpreted information and perceived clinical risk (Chang et al. 2021). Taken together, these qualitative findings suggest that dual‐role assignments can divide attention between clinical surveillance and the complete and accurate transfer of each speaker's message. Role conflict may also arise when the nurse's duty to assess, advise, or intervene competes with the interpreter's duty to reproduce each participant's speech accurately and transparently. More broadly, interpreting can remain institutionally unrecognized or uncompensated labor, while interpreters face a documented tension between professional neutrality and advocacy (Kunreuther and Rao 2023); the nurse's clinical duty of care can intensify that tension.

The ethical risks extend beyond workload. The National Council on Interpreting in Health Care (2005) requires interpreters to maintain confidentiality and impartiality, disclose conflicts of interest, remain within professional role boundaries, and continue to follow all interpreting standards when they hold an additional professional role; the standards explicitly use an interpreter who is also a nurse as an example. In a dual‐role encounter, access to clinical information must not lead to disclosure beyond the treating team, and clinical knowledge must not be used to add, omit, alter, or independently explain either participant's message. Neutrality may also be difficult to maintain—or to demonstrate to the patient—when the interpreter–nurse is simultaneously responsible for care delivery or institutionally aligned with the clinical team. Consent involves two related risks: the patient may not understand or freely accept the dual‐role arrangement, and treatment consent may be compromised if the interpreter–nurse independently explains or promotes the clinician's recommendation. Hsieh and Kramer (2012) document consent‐related tasks being delegated to interpreters and show how such delegation can blur professional boundaries.

Accordingly, before a dual‐role encounter begins, hospitals would need to explain the arrangement, obtain the patient's agreement, and offer access to an independent qualified interpreter. An independent interpreter would be required whenever practicable for informed‐consent, highly sensitive, or contested encounters, particularly when the nurse is involved in obtaining or witnessing consent. Protected interpreting slots would remove the nurse from simultaneous patient‐care assignments, set workload limits, require explicit role‐transition announcements, and provide immediate professional or VRI backup. If urgent clinical duties require the nurse to resume the clinical role, interpreting would stop and be transferred to the backup service. These safeguards are proposed governance requirements for future implementation and require empirical evaluation.

In summary, these proposed mechanisms illustrate how the challenges of dual‐role mediation might be addressed within a formalized clinical career pathway. By combining clinical and linguistic expertise, the model identifies a possible professional trajectory for experienced nurses while foregrounding patient‐safety considerations.

## 5 Conclusion

This study offers a document‐based competency alignment analysis of selected South Korean medical interpreter certification and training documents, situated within the high‐demand yet under‐explored Korean–Russian medical communication context. Through a benchmark‐alignment analysis against international standards, it identifies structural disparities within the selected training and certification documents, complemented by a conceptual Interpreter–Nurse Pathway proposal.

The descriptive mapping yielded a total score of 9 out of 16, equivalent to 56.3% of the theoretical maximum. Three of the eight domains (37.5%) met the Level 2 criterion of explicit linkage to both instruction and assessment: ethics, confidentiality, and neutrality; accuracy, completeness, and terminological competence; and clinical knowledge and medical terminology. The remaining five domains were mapped to Levels 0 or 1. Within the limits of this documentary evidence, these findings support consideration of medical interpreting as a specialized clinical communication service rather than a generic linguistic service.

A key contribution of this work is the transition from identifying documentary gaps to formulating the Interpreter–Nurse Pathway as a conceptual policy and workforce‐development proposal. The pathway outlines how dual‐role practice might be formalized through training, credentialing, and institutional governance and provides a framework for future feasibility assessment and outcome research.

It must be noted, however, that the seven selected documents cannot capture the full breadth of South Korea's national medical interpreter infrastructure, and that the focus on document analysis limits the verification of causal relationships in clinical practice. The alignment reported here is moreover relative both to a benchmark grounded in one national policy framework and to a mapping framework developed and applied by a single researcher, whose repeated coding evidences internal consistency alone. The Interpreter–Nurse Pathway was derived conceptually from the documentary findings and supporting literature and was not implemented or evaluated for feasibility, effectiveness, patient safety, or workforce outcomes. Future research might therefore turn to the question that document analysis cannot itself answer: whether documented linkage between instruction and assessment corresponds to interpreting performance in the clinical encounter.

## Author Contributions

Conceptualization, methodology, data collection, formal analysis, Writing – original draft, and Writing – review and editing: N.Y. Kim.

## Funding

The author has nothing to report.

## Conflicts of Interest

The author declares no conflicts of interest.

## Ethical Approval

No ethical approval was required.

## Data Availability

All source documents analyzed in this study are publicly accessible.
